# A half century of electronic fetal monitoring and bioethics: silence speaks louder than words

**DOI:** 10.1186/s40748-017-0060-2

**Published:** 2017-11-21

**Authors:** Thomas P. Sartwelle, James C. Johnston, Berna Arda

**Affiliations:** 1 Deans and Lyons, LLP, Houston, TX USA; 2 1150 N Loop 1604 W, Ste 108-625, San Antonio, TX 98110 USA; 3 Global Neurology Consultants, Auckland, New Zealand; 4 Department of Medical Ethics, University of Ankara, Ankara, Turkey

**Keywords:** Medical ethics, Cerebral palsy, Electronic fetal monitoring, Medical malpractice

## Abstract

Bioethics abolished the prevailing Hippocratic tenet instructing physicians to make treatment decisions, replacing it with autonomy through informed consent. Informed consent allows the patient to choose treatment after options are explained by the physician. The appearance of bioethics in 1970 coincided with the introduction of electronic fetal monitoring (EFM), which evolved to become the fetal surveillance modality of choice for virtually all women in labor. Autonomy rapidly pervaded all medical procedures, but there was a clear exemption for EFM. Even today, EFM remains immune to the doctrine of informed consent despite continually mounting evidence which proves the procedure is nothing more than myth, illusion and junk science that subjects mothers and babies alike to increased risks of morbidity and mortality. And ethicists have remained utterly silent through a half century of EFM misuse. Our article explores this egregious ethical failure by reviewing EFM’s lack of clinical efficacy, discussing the EFM related harm to mothers and babies, and focusing on the reasons that this obstetrical procedure eluded the revolutionary change from the Hippocratic tradition to autonomy through informed consent.

## Background

“Declare the past, diagnose the present, foretell the future. As to diseases, make a habit of two things—to help and not to harm.” From the book *Epidemics I*

Fifty years ago electronic fetal monitoring (EFM) became an overnight sensation when first introduced into labor suites. Declared to be the long awaited cerebral palsy (CP) cure [1], EFM, in a very short time, was virtually the sole monitoring method used in the industrialized world, the *raison d’etre* of fetal surveillance in labor [2–5]. And today EFM remains obstetrics’ *deus ex machine* [6] despite overwhelming and damning scientific evidence that EFM theory is nothing more than myth and wishful thinking [7–18] and despite evidence that CP is caused chiefly by prenatal factors operating before labor begins, that CP is not preventable by any response to EFM patterns, that EFM has never reduced the rate of CP in 50 years of intense use and supposed improvement [4–13, 15–31], and that EFM use has and is today causing more harm to mothers and babies that it has ever helped [5, 8, 9, 12, 13, 17].

At almost the same instant that EFM became a clinical sensation, bioethics, a word coined in the 1960s, became a recognized entity, linking scientific advances to human values [32]. The foundation of bioethics was autonomy—a person’s right to choose or refuse medical treatment based on informed consent—replacing the centuries-old deontology based on the Hippocratic tradition of benign paternalism—the physician’s duty is to choose the treatment for the patient [32–35].

Linking EFM use with the new bioethics should have been a seamless affair following Thomas Kuhn’s six stages of scientific revolution resulting in his now famous paradigm shift from the old to the new [36]. And while this shift to the new bioethics indeed occurred in most medical arenas [32–35], it failed utterly with EFM and obstetrics. Today obstetrical EFM use without mothers’ informed consent is the epitome of medical paternalism. The cause of this momentous failure was the confluence of three powerful influences that even the brilliant Kuhn failed to see on the horizon.

Throughout EFM’s rise to deus ex machina and its evasion of autonomy-informed consent, the clinical evidence against EFM rose up like a mammoth volcanic ocean island, plain for all to see [5–31]. And while a few clinicians were bold enough to challenge the EFM medical establishment that insisted on continued EFM use despite the evidence of harm, evidence that should have provoked bioethical outrage, the bioethics community’s silence was deafening and remains so today. The question is, why the silence?

And just as important a question is, could bioethics, having no enforcement mechanisms other than words, forced the EFM establishment to recognize autonomy-informed consent and rescued those mothers who believed in EFM’s magic powers only because they were never told the truth about EFM? The answer is yes. As will be demonstrated, bioethical silence is louder that its words, but if words had at least been spoken and written bioethics could have changed forever the EFM dynamic just as Kuhn foretold.

## In the beginning

“To help and not to harm” has been an integral part of medicine from the very first attempts to organize medicine into books and explain theories of disease and the moral, ethical wisdom associated with healing and healers [32]. The thought is perhaps more familiar when written, “first do no harm,” and while both phrases have obscure origins, they have been repeated as part of the Hippocratic tradition by many Western physician commentators and teachers throughout the centuries [32–34, 37]. Repeated not only in writings and lectures, but also formalized in various ethics codes like Percival’s Code, the codes of early medical societies, the first AMA Code in 1847, and just about all subsequent ethics codes of Western-oriented medical societies [32]. It is curious, however, why it was necessary to warn physicians not to harm their patients when the idea of helping patients is dominant. The idea of doing no harm, however, becomes apparent when the second Hippocratic medical principle is considered—it is the physician who must decide what is best for the patient according to the physician’s ability and judgment [32–34].

Thus, Western ethics through the ages was dominated by Hippocratic medical paternalism—physician-centered medicine [38]. Oliver Wendell Holmes, addressing an 1871 medical school graduating class, advised the physicians to conform to the Hippocratic tradition and conceal almost everything from patients, saying that the patient had no right to the truth [38]. In the 1930s, when a patient asked the physician a question during an examination, the physician “slapped her face, saying, ‘I’ll ask the questions here. I’ll do the talking.’” [38] In 1944, President Roosevelt’s physicians did not reveal to him their diagnosis of elevated blood pressure and congestive heart failure diagnoses, a year before a stroke took his life [39]. In 1961, a published study revealed that 88% of U.S. physicians surveyed did not tell the truth to terminally ill cancer patients [33, 38]. And it was not until 1981 that AMA revised its Principles of Medical Ethics to revise the previously accepted Hippocratic-approved lying to patients, mandating that physicians were required to deal honestly with patients and colleagues [33].

This was the state of the centuries-old medical ethics milieu in 1960, when, amid unimagined breakthroughs in science, medicine, and technology, medical inventors worked to perfect an electronic fetal monitor that could, so they believed, predict fetal distress and prevent cerebral palsy [1, 2]. It was a milieu in which physicians were viewed by themselves and the public as being superior to patients and most others in knowledge and insight. Physicians were authoritarian, determining what was in the patients’ best interests, even if the patients did not agree [34].

## Deus ex machina

Thus, in 1970, when EFM was introduced into clinical practice [2], obstetricians were comfortable operating in the Hippocratic sphere in which they made all medical decisions without input from patients. There was, therefore, no necessity to inform patients that EFM was totally experimental and had never been proven in even one clinical trial [5–17]. Nor was there any necessity to advise patients that the very foundational theories undergirding EFM—fetal heart rate reflected past and present fetal brain function and oxygen deprivation in labor was the sole cause of CP—were unscientific myths handed down from generation to generation and untested by modern medicine [4, 5, 7–13, 15–18, 20–28]. Rather, obstetricians throughout the industrialized world accepted the new monitor as the machine that would, as its proponents assured the world, reduce by half intrapartum deaths, mental retardation, and CP [1]. There was no necessity for informed consent, because that concept is a product of bioethics, which, in 1970, had only just emerged as a deontology that would eventually overtake the Hippocratic tradition [32–34]. While simple consent—the patient may say “yes” or “no” to a surgery—was a legal concept [38, 40], true informed consent, the product of bioethics and autonomy [33–35], lay in the future. Thus, when it came to employing the new EFM, obstetricians continued in the Hippocratic physician-is-dominant tradition. And, unfortunately, as will be seen, obstetricians and EFM have continued in the Hippocratic tradition despite the fact that overwhelming evidence demonstrates EFM use has done more harm than good for mothers and babies during the last 50 years.

## A new deontology

Immanuel Kant is credited with the development of deontological ethical theory, a result of enlightenment rationalism and liberal political philosophy, the central focus of which is the individual’s liberties and rights [33]. This liberal political philosophy bypassed Western medicine, however, until after World War II, when a series of disclosures, beginning with the Nuremburg trials and disclosure of wartime medical experiments, began to focus attention on medical research conducted without patients’ consent [33, 34, 41]. The Tuskegee syphilis study, plutonium injections by Manhattan Project doctors, radioactive iodine trials, and a host of other human experiments, 22 of which were exposed in Henry Beecher’s 1966 groundbreaking whistleblower exposé, shocked the public [41]. The common denominator of these medical experiments was that meaningful consent was never obtained from the patients [38, 41].

These experiments and their public exposure, along with other societal upheavals in the 1950s and ‘60s, caused physicians, scientists, philosophers, religious and legal scholars, and other disciplines, to come together to focus on the moral, ethical, and philosophical ambiguities presented by the dizzying array of worldwide scientific medical advances [32–35, 38]. This new discipline, said to have been born around 1970 [32–34], soon took on a new name—bioethics.

This new deontology, based on duty, was dominated by one overwhelming theme—autonomy—respect for the individual and individual self-rule, a duty, fidelity, and faithfulness of one human being to another [34], such that the individual patient could meaningfully choose her medical treatment based on knowledge of the choices available inputted by the physician. And while the term “informed consent” had been used before bioethics was born, from 1970 on, that term was inculcated with new meaning, becoming synonymous with autonomy and driving medicine’s focus away from Hippocratic physician-centered medicine to patient-centered medicine, where it remains today [32–34, 38]. Closely associated with this first bioethics principle are two duties—non-maleficence—do not impose unnecessary harm or risk of harm—and beneficence—dedication to the patient’s welfare, a positive medical goal different in substantial degree from merely avoiding harm [32–35, 38].

While there are still many controversies over the exact meaning and reach of these and other bioethical duties and obligations, it is clear that medicine’s primary bioethical, moral duty is to enable patients to make their own medical decisions, perhaps even wrong in the physician’s judgment, decisions based on relevant, current information provided by the physician [32–35, 38]. And while it is assumed that a given number of patients will not want the burden of deciding, that does not diminish the physician’s duty to provide the information to all patients—in other words, meaningful, informed consent is a moral imperative [33, 34]. This imperative and much of bioethics was, of course, the antithesis of the Hippocratic traditions that had dominated medicine for centuries, yet bioethical thinking became dominant in medicine in only a few short years. A paradigm shift occurred in almost all aspects of medicine, save and except one—electronic fetal monitoring.

## The revolution

Science is always progressing. New theories become settled science and old theories disappear. Ptolemy’s math was impeccable. His calculations were subject to multiple proofs by scholars for well over a thousand years. The conclusion was the same—the earth was the center of the universe. Not so, said Copernicus. Geocentricity was a myth. Real-world cosmology was heliocentric—the sun was the center of the universe. There were few believers. The myth had stood the test of time. One hundred years later, Galileo confirmed the new cosmology with a telescope.

The Copernican revolution was Thomas Kuhn’s prime example of how science is driven to change [36]. In normal periods of science, a paradigm exists by which certain anomalies can be solved. But a crisis in science arises when confidence is lost in a paradigm’s ability to solve particularly worrisome anomalies, and thus a scientific revolution occurs by which an existing paradigm is superseded by a rival.

Before Kuhn, there was little to explain how science changes. There was, however, a conception of scientific change as a smooth, flowing addition of new truths to the stock of old truths and an occasional correction of past errors. According to Kuhn’s theory, science is not uniform, but has normal and revolutionary or extraordinary phases, which he referred to as the puzzle-solving power of the competing ideas. But these competing theories, according to Kuhn, are generally incommensurable, because they share very little commonality [36]. And so it was that Kuhn’s scientific revolution perfectly presaged the medical ethics revolution from the Hippocratic paradigm to the bioethics paradigm.

As we have seen, the bioethics paradigm shift occurred when the Hippocratic ethics was found inadequate to solve the exposés related to medical human experimentation without consent, as well as other patient centering problems [32–35, 38]. The change from Hippocratic ethics to bioethics was extremely rapid when compared to Kuhn’s Copernican revolution and other examples of scientific paradigm shift, but the paradigm shift to bioethics did indeed follow Kuhn’s theory. Autonomy replaced physician-centered medicine, hospital ethics committees were created, institutional review boards were appointed, and many other dynamic changes occurred in the practice of medicine and the field of medical scientific research [33–35]. But while many things changed, at least one thing stayed the same—electronic fetal monitoring, an experimental machine, is still used today without the informed consent of mothers and with no explanation of the conflict of interest physicians have with respect to continued EFM use despite a half-century of uncontradicted proof that EFM does more harm than good.

## Is EFM really experimental?

EFM began clinical life with only a few detractors questioning its basic premise [42]—the conventional but unproven wisdom that CP and neurologic birth maladies were caused by birth asphyxia, hypoxia, and cerebral ischemia, which were reflected in out-of-norm fetal heart rate that, if stopped in time—by C-section or instrumented delivery—would prevent CP and birth maladies [4, 5, 7–11]. Most physicians simply accepted EFM inventors’ claims and promises, even though EFM inventors skipped the rigors of the scientific method and the crucible of randomized clinical trials, and introduced EFM with no instruction manual, with unrealistic efficacy expectations, and no clearly defined parameters for use [43]. As EFM accelerated in use to 85% of births in the United States [5], more and more vehement criticism appeared following its first clinical use. Criticism completely ignored by most physicians and their birth related professional organizations (BRPOs) worldwide as well as ethicists [5, 7–9, 25].

When EFM was subjected to 12 clinical trials versus auscultation beginning in 1976, EFM showed no benefit, but only a dramatic increase in C-sections due to EFM’s 99% false-positive rate [7, 11, 19, 26–29]. In the 1970s, critics wrote that EFM use is unjustified by any evidence [44, 45]. In the 1980s, critics wrote that EFM is increasing unnecessary C-sections [46], and the procedure should be abandoned [46]. In the 1990s, critics wrote that because of EFM and medical malpractice liability concerns, United States obstetricians were performing cesarean sections at a rate much higher than anywhere else in the world; [47] EFM is nothing but a disappointing story, and the hoped-for EFM benefit has not been realized; [48] EFM promised much but achieved little because fetal heart rate changes reflect neurologic insult earlier in pregnancy, not intrapartum events; [49] and no data exists that intervention based on any EFM patterns has reduced CP [49]. By the turn of the century, the critics became even more vocal as the evidence proved CP was not caused by birth events and EFM was not in any manner efficacious, but indeed harmful, primarily because it triggered unnecessary C-sections with the attendant morbidity and mortality: tests leading to unnecessary abdominal surgery in 99% of cases is absurd; [17] EFM has done more harm than good to mothers and babies; [17] EFM has had no effect on perinatal mortality or neurologic morbidity; [26] EFM interpretation is subjective, difficult to standardize, and poorly reproducible; [6] after almost 50 years, there is no consensus on EFM pattern interpretation and management; [3] EFM as a screening tool for absence of harm is no better than tossing a coin; [12] EFM overall caused more harm than good; [12] there is a growing consensus in the maternal-fetal medicine community that it is time to start over with EFM and establish a common language, standard interpretation and management principles [43]. In 2014, the authors of *Neonatal Encephalopathy and Neurologic Outcome, Second Edition*, published by ACOG and American Academy of Pediatrics, and assisted by an international array of Task Force consultants, conceded EFM defeat after a concerted 50-year effort to make EFM predict asphyxia, hypoxia, ischemia, acidemia, CP, and neurologic injury: “There are no long-term benefits of EFM as currently used” [21] (at 88); “no evidence exists demonstrating that electronic FHR monitoring reduces the rate of neonatal encephalopathy” [21] (at 92); “there is no evidence in the current literature to support the ability of practitioners to predict neonatal neurologic injury, cerebral palsy, or stillbirth using EFM” [21] (at 91); “cesarean delivery as an obstetrical intervention to reduce neonatal encephalopathy and cerebral palsy has been considered unsuccessful” [21] (at 104). This Task Force report was endorsed not only by ACOG and AAP, but also by 11 other worldwide birth-related organizations from Australia, New Zealand, Japan, Canada, and the United States [21].

Even today, efforts to make EFM heel to command have failed once more. EFM assisted by ST-segment analysis did not improve perinatal outcomes or decrease C-section rates [50], which are now 33% in the United States [51] and even higher in other parts of the world [5]. And more voices are calling on BRPOs to condemn EFM use in both labor rooms and in CP-EFM worldwide litigation because it is junk science [5, 7–13, 15–17, 19, 25, 43, 52]. Other voices are calling to reduce the C-section rate recognized to be caused in large part by defensive medicine and EFM’s 99% false positive rate [12, 53, 54], which subjects mothers and babies to unnecessary morbidity and mortality risks from not only the immediate surgery but from subsequent sequela [5, 8, 9, 12, 13, 17]. And other voices are citing increasing evidence that C-sections are exposing babies to the specter of subsequent lifelong chronic diseases and neuropsychiatric disorders [55–58].

A practical, common sense definition of experimental medical procedures would be that until the published, peer-reviewed medical evidence regarding risks, benefits, and overall safety and efficacy demonstrate that the procedure has a beneficial effect on healthcare outcomes, the procedure is experimental. It is without question that the unrefuted evidence over five decades has proven EFM is unsafe, un-efficacious, and has no beneficial effect on healthcare outcomes. Just the opposite. EFM does little good, but considerable harm. Thus, it is unfathomable that the ACOG-AAP Task Force [21] and individual practitioners in many countries continue to call for EFM use in every labor [2, 59–61].

Why?

## Vincible ignorance

It is no longer a secret why BRPOs, medical societies, and individual physicians and hospitals ignored the 50-year EFM volcano erupting with undeniable, unrefuted evidence that EFM is junk science and causes harm to mothers and babies. The secret has been exposed in government studies [62, 63], medical journals [4, 7, 9, 11, 12, 15, 16, 19, 23, 43, 47, 53, 54], legal journals [5, 25, 64–67], newspaper and popular magazine articles [68, 69] since at least 1979: obstetrical defensive medicine. Physicians and hospitals use EFM because they believe it protects them from trial lawyers and CP lawsuits. This concept of EFM providing malpractice protection is, and always has been, another birth myth, as has been pointed out in the medical and legal literature for many years [5, 8, 25, 52, 64–66]. But, like the other birth myths, the truth would destroy the collective obstetrical illusions handed down from generation to generation. But clinging to obstetrical illusions and myths is merely practicing nineteenth century medicine, which was based on whim, personal belief, and bias, rather than evidence-based, scientific-based medicine. It also violates every past and current concept in bioethics, especially the heart and soul of bioethics—autonomy.

So the question arises, why did bioethics issue an ethical pass to EFM? How did virtually all of medicine succumb to Kuhn’s paradigm shift from Hippocratic ethics to bioethics in just a few decades, while EFM continues to be used without informed consent, continues to cause harm without discernible benefits, and all without raising any concern or alarm from the bioethics community? Who issued EFM the ethics pass?

## In plain sight

Although it is impossible to determine the moment that paternalism died and autonomy was born, we can isolate one period in early EFM history when autonomy was recognized as dominant over paternalistic EFM use. That dominance quickly faded, however, because of influences unforeseen and unaccounted for by contemporary scholars—trial lawyers, medical malpractice lawsuits, and defensive medicine.

In the 1970s, NIH organized consensus-development Task Forces to address recent developments in medical research and practice [62]. One Task Force on predictors of intrapartum fetal distress was composed of physicians, sociologists, lawyers, and ethicists, among others. The Task Force’s 1979 report was subsequently published in three prominent journals [62]. With respect to EFM, the Task Force recommended, because there was no evidence of EFM efficacy, that mothers be informed, during the course of prenatal care and again on admission to the labor suite, about EFM limitations and risks [62]. This Task Force’s EFM bioethical recommendation was followed by the International Federation of Gynecology and Obstetrics and Family Health International (FIGO) in 1986 in their Guidelines for Fetal Monitoring Use: “Mothers should have the opportunity to discuss the use of electronic fetal heart rate monitoring during antepartum care and again upon admission to hospital in labor, so that they are able to give or to withhold informed consent.” [70].

But informed consent never happened. With these two exceptions, EFM medical literature and BRPOs are completely silent regarding when, how, and what to tell mothers about EFM’s limitations and risks and, as the evidence increased in later years, about EFM’s morbidity and mortality, current and future. There is also complete silence in the bioethical literature urging BRPOs and physicians to make EFM informed consent a duty. The EFM informed consent issue did not escape the nursing, midwife, and legal literature [65, 66, 71, 72], and, in fact, is still discussed today [67, 73, 74]. But these voices have been ignored by obstetricians and ethicists alike.

## Murphy’s law and paradigm shifts

Kuhn started challenging the scholarly community with his revolutionary science, paradigms, and puzzle-solving in his 1962 book, *The Structure of Scientific Revolutions* [36]. While Kuhn’s revolution model perfectly presaged the coming bioethical paradigm shift, a retrospective view reveals that Kuhn, his proponents, and critics alike overlooked a paradigm shift taking place under their feet—trial lawyers, medical malpractice, and defensive medicine.

In the 1960s, for reasons still debated today, there was a sudden, dramatic rise in the frequency and severity of medical malpractice lawsuits and claims, which accelerated rapidly to unprecedented levels, precipitating the first of many medical malpractice-insurance coverage crises early in the 1970s [5, 8, 9, 25]. Obstetrics was a particular target because, until EFM, the only evidence of fetal heart rate during labor had been the obstetrician’s recollection. With EFM, there was a permanent paper tracing that trial lawyers’ courtroom experts could interpret years and even decades after a birth and, of course, inevitably find the precise moment the inattentive or ignorant attending physician should have performed a C-section to save what the courtroom experts said was a neurologically perfect infant from lifelong crippling injuries. EFM, nobly conceived, met Murphy’s Law, and for 50 years has been the trial lawyer’s weapon to extract billions from physicians and hospitals the world over, even as the evidence mounted that EFM did not predict CP and CP was not caused primarily by asphyxia, anoxia, hypoxia, or ischemia during birth. On the other hand, physicians, new at the litigation game, ironically adopted the trial lawyers’ preferred weapon, EFM, as their sole CP-lawsuit defense [5, 8, 9, 25]. But over the past half-century, physicians worldwide have lost the CP-EFM battle to the trial lawyers for a variety of reasons [5, 8, 9, 25] and are now engulfed in an epidemic explosion of malpractice lawsuits, especially CP-EFM neurologic impairment birth-injury lawsuits [5, 7–11, 15, 25].

The result of the initial trial lawyers versus doctors litigation battle was defensive medicine—prophylactic medicine of little use to the patient, administered primarily for the protection of doctors and hospitals from trial lawyers and lawsuits [5, 7–11, 23, 25]. And while most defensive medicine received considerable criticism from bioethicists, economists, and some clinicians as being medically and ethically questionable, not to mention costly to society [75–78], the EFM criticism, as we have seen, came only from a small number of clinicians and scholars, and even they never addressed the bioethical issue of physicians violating patient autonomy by EFM use without informed consent. Ethicists defaulted in the EFM debate by their overwhelming silence. The use of a scientifically bankrupt machine solely to protect healthcare providers from trial lawyers and lawsuits when the machine is known to be harming mothers and babies is an egregious conflict of interest and outrageous endorsement of obstetrical defensive medicine—post-modern ethical relativism solely to benefit healthcare providers—and is undeniable proof that evidence-based standard of care and bioethical principles are nothing more than empty rhetoric [79, 80].

A part of bioethical morality requires that patients be allowed autonomy and that in the treatment process the physician refrain from harm to the extent practical. But the third principle—beneficence—requires more than avoiding harm. It requires positive steps to benefit the patient, steps that balance benefits, risks, costs, and other patient goals to produce the most optimum results possible [33, 34]. In other words, beneficence requires the physician to favor the patient’s interest over the physician’s self-interest [33, 34]. This obligation is fidelity. It results in a fiduciary relationship between patient and physician [33, 34]. In daily practice, this fiduciary relationship is often challenged by potential divided loyalties—conflicts of interest—to the physician’s colleagues, institutions, third parties, funding sources, and the physician’s self-protection interests [33, 34]. Conflicts of interest have received enormous attention, primarily due to perceived conflicts in commercial, manufacturing, and financial relationships [33, 34]. However, the EFM conflict of interest—using a device that actually causes harm to patients primarily to protect doctors and hospitals from CP lawsuits—is enormously more compromising than gifts, trips, and money, because it has been and is hidden from public view and because the EFM device is fraudulently presented to mothers as a safety device necessary for a healthy baby. Fidelity could not possibly be more compromised.

Ethicists’ silence on a woman’s right to EFM truth and her right to choose what happens to her body and to her baby has spoken more loudly than words ever could.

## Conclusion - *POST TENEBRAS LUX*

Law and bioethics are separate, distinct entities. Law is more structured, concerning itself with rules that stabilize society’s social institutions and structures. Law concentrates on the criminal and civil penalties to be brought to bear on miscreants who fail to conform [33, 34]. Bioethics, on the other hand, are conceived of as moral rules and ideals—how one ought to act toward others—some aspirational and some even unobtainable. How seriously these moral rules must be taken is still in dispute among the ethical legalists and ethical antinomianists [33, 34], but neither extreme in that debate contradicts the fact that ethical rules are essentially unenforceable save for the mild, occasional organizational enforcement and perhaps occasional licensing rebuke.

But perhaps bioethics’ unenforceability is actually an illusion. After all, at the dawn of bioethics there was no enforceability other than words and public exposure of medical procedures that most agreed were morally wrong and dismissive of individual autonomy. Bioethics changed the entirety of medicine and the Hippocratic tradition that had ruled for centuries. Words were the only weapons needed. So the question arises, can words still be used to change the EFM bioethics? Said another way, can bioethics shed light in the darkness of 50 years of EFM paternalism? It would be a far better thing to voluntarily come to the light of EFM autonomy-informed consent now, rather than being forced into the light tomorrow by the trial lawyers.

## Abbreviations

BRPO: Birth related professional organizations
CP: Cerebral palsy
EFM: Electronic fetal monitoring
